# Effects of Elevated CO_2_ on Photosynthetic Accumulation, Sucrose Metabolism-Related Enzymes, and Genes Identification in Goji Berry (*Lycium barbarum* L.)

**DOI:** 10.3389/fpls.2021.643555

**Published:** 2021-03-11

**Authors:** Yaping Ma, Yun Xie, Rong Ha, Bing Cao, Lihua Song

**Affiliations:** ^1^School of Agriculture, Ningxia University, Yinchuan, China; ^2^College of Forestry, Nanjing Forestry University, Nanjing, China

**Keywords:** *Lycium barbarum* L., photosynthesis, sucrose metabolism-related enzymes, subcellular localization, gene expression patterns

## Abstract

Goji berry (*Lycium barbarum* L.) exposure to elevated CO_2_ (eCO_2_) for long periods reduces their sugar and secondary metabolite contents. However, sugar accumulation in fruit depends on photosynthesis and photoassimilate partitioning. This study aimed to explore photosynthesis, sugar content, and sucrose metabolism-related enzyme activities in goji berry leaves and fruits under ambient and eCO_2_ levels, and identify the genes encoding *L. barbarum* acid invertase (*LBAI*), *L. barbarum* sucrose synthase (*LBSS*), *L. barbarum* sucrose phosphate synthase (*LBSPS*), and *L. barbarum* neutral invertase (*LBNI*), based on transcriptome profiling. Further, the characterization of four identified genes was analyzed including subcellular localization and expression patterns. In plants grown under eCO_2_ for 90 or 120 days, the expression of the above-mentioned genes changed significantly as the photosynthetic rate increased. In addition, leaf and fruit sugar contents decreased, and the activities of four sucrose metabolism-related enzymes increased in leaves, while acid and neutral invertase increased in fruits. Protein sequence analysis demonstrated that *LBAI* and *LBNI* contain a conservative structure domain belonging to the glycosyl hydrolases (Glyco_hydro) family, and both *LBSS* and *LBSPS* belonging to the sucrose synthase (Sucrose_synth) and glycosyltransferase (Glycos_transf) family. Subcellular localization analysis showed that *LBAI*, *LBNI*, and *LBSS* were all located in the nucleus, plasma membrane, and cytoplasm, while *LBSPS* was located in the plasma membrane. The expressions of *LBAI*, *LBSPS*, and *LBNI* were high in the stems, whereas *LBSS* was predominantly expressed in the fruits. Our findings provide fundamental data on photosynthesis and sugar accumulation trends in goji berries under eCO_2_ exposure.

## Introduction

Goji berry (*Lycium barbarum* L.) is a deciduous perennial shrub of the Solanaceae family, traditionally used as a medicinal plant in East Asia (Amagase and Farnsworth, 2011; Pedro et al., 2018). The species has been planted in China for over 2,000 years and contains various bioactive and pharmacological components that promote metabolism and help control diabetes, regulate immunity, and protect the nervous system. Hence, it is widely used for its unique efficacy in immune regulation and anti-aging and anti-tumor effects. Studies have demonstrated that the whole goji berry plant (fruits, roots, leaves, bark, and flowers) contains a variety of phytochemical components, such as polysaccharides (LBP), flavonoids, carotenoids, alkaloids, amides, peptides, anthraquinones, sterols, natural acids, and glycolipids that have high medicinal values and plays a role in functional food development (Wang et al., 2015; Byambasuren et al., 2019). Goji berry fruits contain certain bioactive secondary metabolites which are often used for a wide range of pharmacological and therapeutic purposes as they are thought to have immunomodulatory, anti-inflammatory, anti-mutagenic, anti-cancer, anti-radiation, anti-aging, anti-stress, cardio-protective, and wound healing effects (Leontopoulos et al., 2017a,b; Kwok et al., 2019). Its leaves are also rich in various bioactive compounds, which are used as enzyme inhibitors and for their antimicrobial effects (Pereira et al., 2019; Zhao et al., 2019). Large quantities of dicaffeoylspermine/spermidines have been found in their roots and are thought to have beneficial effects on fever, night sweats, anxiety, kidney problems, backaches, and insomnia (Yao et al., 2018). Hence, as a medicinal and food plant, goji berry has attracted increased global attention and, as such, has broad development prospects in the fields of medicine and nutritional health.

Global warming is rapidly becoming a major threat to water resource availability and plant growth and fecundity worldwide (Kadiyala et al., 2015). Furthermore, rising atmospheric CO_2_ concentrations caused the greenhouse effect are increasingly acknowledged as the primary factor accelerating global climate change (IPCC, 2014). Concomitantly, as CO_2_ is the main reactant in photosynthesis, changes in ambient CO_2_ concentrations directly impact plant physiology and growth (Wei et al., 2018). Plant exposure to short-term elevated CO_2_ (eCO_2_) levels (700 μmol mol^−1^) significantly increases aboveground biomass and grain yield components while having a modest influence on the biochemical composition of the mature grain (although a reduction in plant nitrogen content is concomitant; Soba et al., 2019). In goji berries, results from experiments which simulated eCO_2_, higher temperature, and drought stress conditions associated with climate change showed that these factors significantly affected physiology, important pharmacological secondary metabolites, and sugar accumulation and distribution (Cao et al., 2011; Zhao et al., 2015b; Ma et al., 2019a). Our previous research showed that eCO_2_ increases photoassimilate partitioning and the transport of products derived from photosynthetic metabolisms, such as soluble sugars and polysaccharides, toward stems and leaves (Cao et al., 2011; Hou and Cao, 2011). Furthermore, long-term eCO_2_ decreases net photosynthetic rate (*Pn*), stomatal conductance (*g_s_*), and photosynthetic CO_2_ assimilation rate, while promoting the accumulation of aboveground biomass to a greater extent than that of underground biomass (Guo et al., 2019). Moreover, long-term eCO_2_ causes a clear reduction in fruit sugar content and markedly alters the activities of the sucrose metabolism-related enzymes such as sucrose synthase (SS), sucrose phosphate synthase (SPS), neutral invertase (NI), and acid invertase (AI; Liu et al., 2016; Ha et al., 2019). In addition, eCO_2_ clearly impacts sugar metabolism gene expression. Using transcriptome analysis, the *L. barbarum* UDP-glucuronate 4-epimerase (*LBGAE*), *L. barbarum* alpha-galactosidase (*LBGALA*), and *L. barbarum* malate synthase (*LBMS*) genes, involved in the regulation of four sugar metabolism pathways, were found to be highly differentially expressed under eCO_2_ (Ma et al., 2019a, 2021). Several studies have found that the genes *L. barbarum* acid invertase (*LBAI*; Wang et al., 2014), *L. barbarum* neutral invertase (*LBNI*; Wang et al., 2019), *L. barbarum* sucrose synthase (*LBSS*; Wang et al., 2019), and *L. barbarum* sucrose phosphate synthase (*LBSPS*; Wang et al., 2013) play key roles in sugar transport and regulation in goji berries. However, no study has investigated how these genes regulate sugar metabolism functions or physiology in response to changing environmental conditions, such as climate change.

Our previous study reported that total sugar and carotenoid contents decreased in goji berry fruits after 120 days of growth in an eCO_2_ treatment. Three genes (*LBGAE*, *LBGALA*, and *LBMS*) were identified to play critical regulatory roles in the sugar metabolic pathway at various eCO_2_ concentrations (Ma et al., 2019a). Flavones, carotenoids, and various sugar components are the most important nutritional and medicinal components in goji berries, concentrations of which are determined by a key metabolite, sugar. As a non-referenced genome species, there are no studies on goji berry that clearly outline how gene function is regulated in sugar metabolism. Our work aimed to identify key genes related to sugar metabolism for the eventual goal of identifying and potentially developing goji berry varieties that produce larger quantities of sugar and secondary metabolites in eCO_2_ conditions. Therefore, in this study, we identified sucrose metabolism-related genes *LBAI*, *LBSS*, *LBSPS*, and *LBNI* based on recent studies and analyzed their characteristics, subcellular localization, and temporal and spatial expressions. Additionally, we investigated the photosynthetic characteristics, contents of different sugars, and activities of the sucrose metabolism-related enzymes (LBSS, LBSPS, LBNI, and LBAI) in both leaves and fruits. Our study provides novel insights into the sugar metabolism responses of goji berry to changes in CO_2_ concentrations. Such information is valuable, given the increasing importance of goji berry for medicinal and nutritional uses and the impending plant growth issues associated with climate change. Our study also provides direction for selecting candidate genes that contain high sugar and secondary metabolites contents to use in goji berry breeding.

## Materials and Methods

### Plant Material and Elevated CO_2_ Treatments

Goji berry plants from the cultivar “Ningqi 1” were provided by the Ningxia Academy of Agriculture and Forestry Sciences (Yinchuan, Ningxia, China), and the materials were of the same with *de novo* transcriptome materials (Ma et al., 2019a, 2021). The experimental farm is located in Yongning County in the Yellow River alluvial plain in central Ningxia Province, China (38°13'50.34'' N; 106°14'22.19'' E; 1116.86 m a. s. l.; inland, in the northwest). The climate at the study site is arid and moderately temperate, with a frost-free period of 140–160 days, and annual precipitation of 180–300 mm.

The experiments were conducted in a total of nine open-top chambers (OTCs) with three treatments: ambient (380 ± 20 μmol mol^−1^ CO_2_, considered consistent with the local environment), elevated I (570 ± 20 μmol mol^−1^ CO_2_, predicted concentration for the year 2025), and elevated II (760 ± 20 μmol mol^−1^ CO_2_, predicted concentration for the year 2050). CO_2_ concentration settings were based on projections reported in the IPCC report (IPCC, 2014). Each treatment was performed in three OTCs (nine chambers in total) between May 1, 2019 and October 1, 2019.

Nine 1-year-old goji berry cuttings were planted evenly in each OTC and grown uniformly. The growth management of the plants was as follows: organic fertilizer (organic content ≥45%, total content of N, P, and K ≥ 5%, water ≤ 30%, PH:5.5~8.5; according to the national standard of organic fertilizer NY525-2012) was applied in each OTC in April, and organic water-soluble fertilizer (18-18-18+2MgO Multi-feed, Haifa, Israel) was applied in July, August, and September. During the goji berry growing period, 1.5% *Sophora flavescens* lectin and 0.9% avermectin were used to control aphids and gall mites.

**Figure 1:**
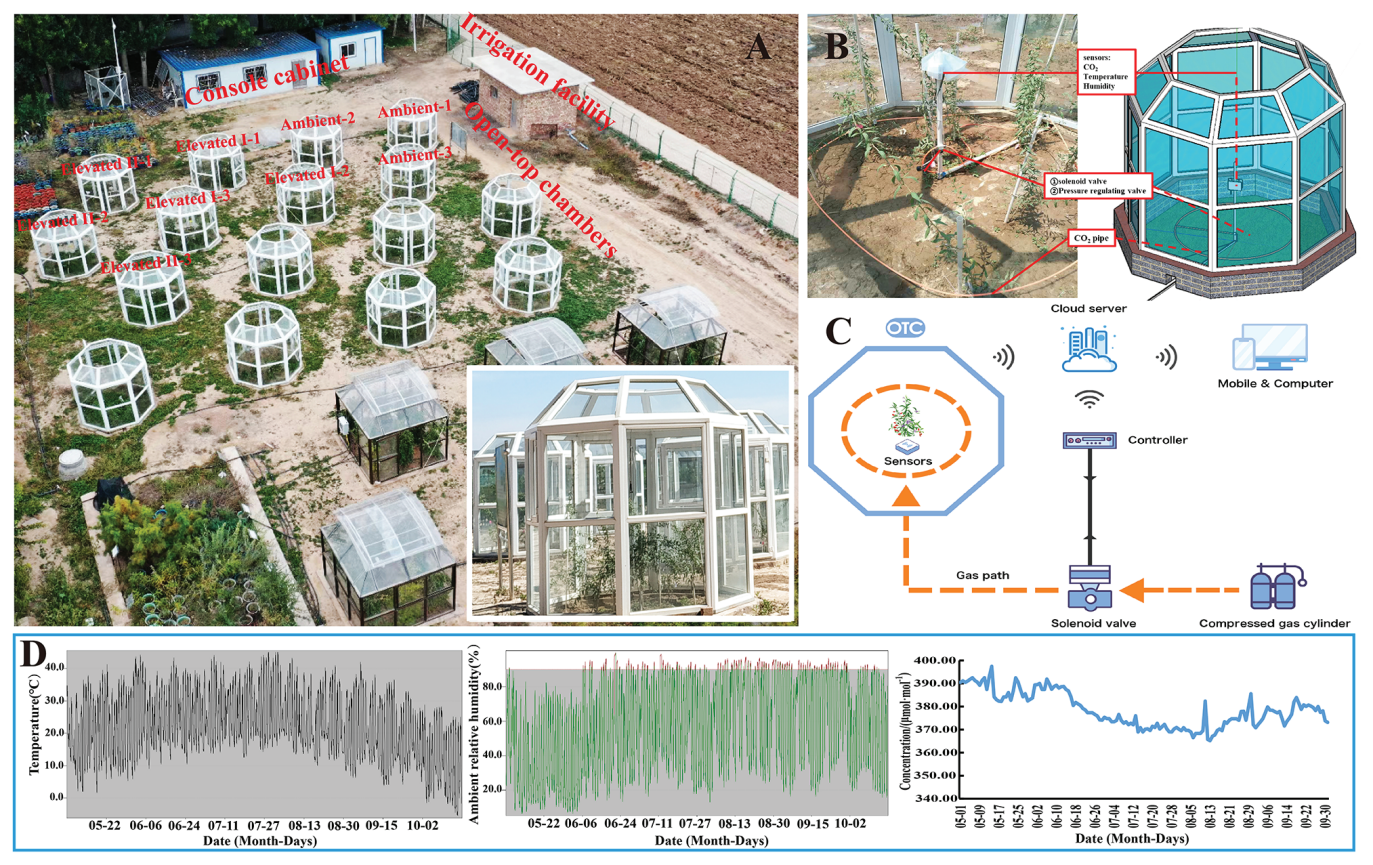
Open-top chamber (OTC) layout **(A)**, chamber structure **(B)**, control system **(C)**, and natural environment temperature, air relative humidity, and CO_2_ concentration monitoring **(D)**.

The OTC control system and experimental layout are shown in Figure 1A. The CO_2_ simulation control system consisted of three parts: the OTC, control system, and monitoring system. The OTC is a regular octagonal prism structure made of plastic, steel, and 4 mm of thick high-transmittance glass with dimensions of 1.08 m in length, 2.78 m in diameter, and an inner and outer height of 2.55 m and 2.10 m, respectively. Each OTC was set up in an interval length of 3 m (Figure 1A). The monitoring system consisted of CO_2_ analyzers (JQAW-12AC, 0–2000 μmol mol^−1^, Beijing Kunlun Coastal Sensing Technology Co., Ltd., Beijing, China), temperature and humidity sensors (JWSK-6ACW, Beijing Kunlun Coastal Sensing Technology Co., Ltd., Beijing, China), and a data acquisition system (Figure 1B). The control system also contained other components, including a programmable logic controller (PLC), GPRS communication module, touch screen, micro-relay, CO_2_ pressure reducing valves, solenoid valves, perforated windpipes, and CO_2_ cylinders. The OTC control system automatically collected and uploaded data every 6 min using a system controller coupled to the proportional-integral controller. A linear controller enabled deviation monitoring according to a given and an actual output value. The system was also equipped with a GSM communication module connected with the PLC through the protocol point to point interface (PPI) and is used to upload all the data to a web server. CO_2_ exposure time was set between 8 am and 8 pm daily. The OTC control and monitoring systems and data were accessed in real-time through a web browser and mobile app, which reduced operating costs and allowed environmental variable monitoring (Figure 1C). The control system, described in more detail by Ma et al. (2019b), aims to assist climate change research and has successfully applied for and is the subject of a patent application (Ma et al., 2020). The variation curves of CO_2_ concentrations, temperatures, and ambient relative humidity monitored in the natural atmosphere from May 1 to October 1, 2019 are shown in Figure 1D; the averages were 378.48 μmol mol^−1^, 21.7°C, and 58.2%, respectively, with ranges of 365.17–397.46 μmol mol^−1^, −5.6 to 45.1°C, and 6.2–99.9%, respectively. Meanwhile, in the OTCs, the average CO_2_ concentrations under ambient, elevated I, and elevated II treatments were 369.33, 545.93, and 751.49 μmol mol^−1^, respectively. The average temperature was 22°C, with a minimum temperature of 15.67°C and maximum temperature of 33.72°C, and the average ambient relative humidity was 62.2%. Ma et al. (2019b) report more detailed OTC monitoring data.

Root, stem, leaf, and fruit tissue samples were collected following 90 (August 8) and 120 (September 7) days of treatment to determine sugar contents and enzyme activities. Enzyme-encoding gene transcript levels were determined using real-time quantitative reverse transcription PCR (qRT-PCR) in fruit and leaf tissue samples. Upon collection, samples were immediately frozen in liquid nitrogen and stored at −80°C until analysis.

### Photosynthetic Rate and Chlorophyll Content Measurements

Net photosynthetic rate (*Pn*), water use efficiency (WUE), intercellular CO_2_ concentration (*C_i_*), stomatal conductance (*g_s_*), and leaf transpiration rate (*Tr*) under ambient and eCO_2_ (I and II) conditions were measured after 90 or 120 days between 9:00 and 11:00 am with a portable photosynthesis system (Li-Cor 6,400; Li-Cor Inc., NE, United States). Measurements were performed after stabilization for 3 min to attain a steady-state under a light-saturating photosynthetic photon flux density of 2000 μmol m^−2^ s^−1^ and a leaf temperature of 28°C. Seedlings with 6–8 leaves from top to middle were selected for measurements. WUE was calculated using the formula WUE = *Pn*/*Tr*. From May 23 to September 22, 2019, at least 10 leaves from top to middle were selected to determine chlorophyll contents at 15-day intervals using a portable chlorophyll meter (*SPAD*-502, Konica Minolta, Japan), which was assumed to reflect relative chlorophyll content.

### Sugar Content and Sucrose Metabolism-Related Enzyme Analysis

Fruits and leaves were collected from trees kept under ambient or eCO_2_ (I or II) conditions in OTCs after 90 and 120 days and stored at −80°C to determine glucose, fructose, starch, and sucrose contents, and the activities of sucrose metabolism-related enzymes LBAI, LBSS, LBSPS, and LBNI. All the below experiments contained three biological replicates, and each replicate included at least three technical replicates.

Glucose, fructose, sucrose, and starch were determined by the anthrone-sulfuric acid method with slight modifications (Leyva et al., 2008; Li et al., 2011). Glucose, fructose, and sucrose: The samples (5 g) were finely grounded using mortar and pestle with 75% ethanol. The mixture and its rinsed components were added to the volumetric flask amounting to a total volume of 80 ml. The solution was placed in an 80°C water bath for 15 min and then cooled down 20°C. Thereafter, it was centrifuged at 8000 rpm for 10 min, of which the supernatant was extracted into 10 ml test tubes with added distilled water to 10 ml. Then, 1 ml of the filtered sample solution was placed in a test tube, and 1 ml of distilled water was added, making a total volume of 2 ml. Using a burette, anthrone-sulfuric acid was added to the solution, shaken for 10 s, and heated in a boiling water bath for 3.5 min. The above steps were repeated with 2 ml distilled water as a control. The absorbance was measured at 620 nm wavelength using a spectrophotometer.

Starch: The samples (5 g) were weighed, grounded, and properly mixed in a mortar, and then transferred to a 50 ml centrifuge tube. To moisten the samples, 2 ml of 80% ethanol was added to the mixture. Thereafter, 5 ml of distilled water and 25 ml of 80% ethanol were added; the solution was thoroughly mixed and placed at 20°C for 5 min, and then centrifuged at 2500 rpm for another 5 min. The supernatant was discarded, and 30 ml of 80% ethanol solution was added. The above steps were repeated to obtain the residue, then 5 ml of distilled water and 30 ml 52% perchloric acid solution were added, and the solution was placed in a vortex oscillator for 10 min. Subsequently, it was centrifuged at 2500 rpm for 10 min, of which the supernatant was transferred into a 100 ml volumetric flask with 100 ml of distilled water. The solution was then filtered, and 10 ml was placed in a 250 ml volumetric flask. After that, distilled water was added, and 2 ml was taken and placed in a 10 ml tube. It was placed in a cold bath for 2 min, and then 6 ml of anthrone sulfate solution was added and thoroughly mixed. It was again placed in a cold bath for 2 min, a boiling water bath for 5 min, and then cooled to room temperature 20°C. Absorptivity was determined at 640 nm.

LBAI, LBNI, LBSS, and LBSPS activities assay was conducted with reference to Zheng et al. (2008) and Liu et al. (2016) with a slight modification.

LBAI and LBNI activities assay: Samples (0.5 g) were finely grounded in liquid nitrogen using a chilled mortar and pestle for 10 min, and extraction buffer [200 mmol·L^−1^ phosphate buffer, 5 mmol·L^−1^MgCl_2_, 0.1% beta-mercaptoethanol, 0.05% Triton-X 100, 0.05% bovine serum albumin (BSA), 2% polyvinylpolypyrrolidone (PVPP), pH 7.5] was added. The homogenate was centrifuged at 20000 rpm for 30 min, and the supernatant was collected. (NH_4_)_2_SO_4_ up to 80% saturation was added and left to settle for 30 min, and then centrifuged at 20,000 rpm for 20 min. The supernatant was removed, and desalting buffer (20 mmol·L^−1^ phosphate buffer, 0.25 mmol·L^−1^MgCl_2_, 0.01% beta-mercaptoethanol, 0.05% BSA, pH 7.5) was added. To dissolve precipitate again, the residue was dialyzed for 24 h at 4°C.

LBSS and LBSPS activities assay: The extraction method was same with the method described above; however, different buffers were used; extraction buffer [200 mmol·L^−1^ HEPES-NaoH, 5 mmol·L^−1^MgCl_2_, 0.01% beta-mercaptoethanol, 0.05% Triton-X 100, 0.05% BSA, 2% PVPP, 1 mmol·L^−1^ ethylene diamine tetraacetic acid (EDTA), 10 mmol·L^−1^ L-Ascorbic acid, 10 mmol·L^−1^ cysteine hydrochloride, 2% glycerol, pH 7.5]; desalting buffer [20 mmol·L^−1^ HEPES-NaoH, 0.25 mmol·L^−1^MgCl_2_, 0.01% beta-mercaptoethanol, 0.05% BSA, 1 mmol·L^−1^ EDTA, 1 mmol·L^−1^ ethylene glycol tetraacetic acid (EGTA), 2% glycerol, pH 7.5].

### Identification, Characterization, Molecular Cloning, Vector Construction, and Transformation of Sucrose Metabolism-Related Genes

According to recent studies, the key genes closely related to sugar transport and regulation in goji berry were isolated using homologous cloning. The functions of *LBAI*, *LBSS*, *LBSPS*, and *LBNI* were predicted (Wang et al., 2014, 2019; Zhao et al., 2015a). Our work also found that *LBGALA*, *LBGAE*, and *LBMS* have key regulatory functions in sugar metabolism (Ma et al., 2019a, 2021). Our work aimed to find genes closely related to sugar metabolism in goji berry and further study their functions in order to improve the breeding of high sugar varieties in the future. Hence, we selected previously identified genes, *LBAI*, *LBSS*, *LBSPS*, and *LBNI*, to analyze their characteristics to provide a basis for further functional studies.

The sequence for *L. barbarum* sucrose metabolism-related genes *LBAI* (GenBank: KM191309.1, KC776575.1), *LBSS* (KC907702.1, KM191310.1), *LBSPS* (KM191308.1), and *LBNI* (KR026955) were searched against the NCBI nucleotide collection (nr/nt) using BLASTN (GenBank; Benson et al., 2012),^1^ and the full-length sequences were downloaded. BLASTN was performed again on each downloaded sequence against the database of the goji berry *de novo* transcriptome (Ma et al., 2019a) using a local database constructed with BLASTN 2.9.0+ (Morgulis et al., 2008). Sequence data identified for sucrose metabolism-related genes *LBAI*, *LBSS*, *LBSPS*, and *LBNI* were deposited into GenBank under reference numbers MN718195, MN718196, MN718197, and MN718198, respectively.

The ProtParam tool was used to analyze protein physicochemical properties.^2^ The open reading frame (ORF) was identified by utilizing an ORF finder.^3^ DNAMAN (V8.0, Lynnon Corporation, Vandreuil, QC, Canada) software was used to analyze the molecular mass, base composition, and base distribution of the nucleic acid sequences, and SignalP4.1 was employed to predict protein subcellular locations. Also,^4^ SWISS-MODEL was used to predict the structure of proteins.^5^

The cloning kit used was the NovoRec® PCR seamless one-step directional cloning kit (Novoprotein Scientific Inc., Shanghai, China). Primers were designed using Primer Premier 6.0 software (Premier Biosoft, Palo Alto, CA, United States). Primer sequences are listed in Supplementary Table S2. According to the designed primer, the target gene was cloned by PCR using sucrose metabolism-related gene sequences as templates. The reaction system was as follows: 25 μl of 2X Fast Pfu Master Mix (E035-02A Novoprotein, Shanghai, China), 0.5 μl of F (10 μM), 0.5 μl of R (10 μM), 0.5 μl of template, and up to 50 μl of distilled and deionized H_2_O. The reaction program for PCR was as follows: 95°C for 5 min, followed by 30 cycles of 94°C for 30 s, 50°C for 30 s, 72°C for 30 s per kb, and 72°C for 8 min. The 1% agarose gel was prepared, and then the PCR product was added to the gel hole, and the gel was placed in the electrophoresis tank. The voltage was set to 60–100 V, and the sample moved from the negative electrode (black) to the positive electrode (red) direction. Electrophoresis stopped when the bromophenol blue moved to approximately 1 cm below the plywood. A fluorescent band displayed the presence of DNA under a UV lamp, and it was photographed this using a gel imaging system. The gel containing the target band was quickly cut under the UV lamp and transferred to a 2 ml centrifuge tube. According to the instructions of the gel recovery kit (Bioteke Corporation, Beijing, China), the target fragment was recovered and stored at −20°C.

The plasmids pART-CAM-EGFP was digested with EcoRI and XhoI, and samples were loaded onto an agarose gel to detect the results. The large segment of the vector corresponding to the gel strip was cut, and a gel recovery kit (Bioteke Corporation, Beijing, China) was used to recover the enzyme cut products. The double-enzyme digestion reaction system used was as follow 8 μl of 2XTango buffer, 1 μl of XhoI, 1 μl of EcoRI, 8 μl of plasmids, and up to 40 μl of distilled and deionized H_2_O. Samples were incubated at 37°C for 2.5 h, followed by 65°C for 20 min until enzyme inactivation. The target DNA fragment and linearized carrier were added to the centrifugal tube with a certain molar ratio (3:1–10:1) for recombination reaction. After mixing at 37°C for 30 min, there was an immediate transformation, and the remaining sample was stored at −20°C.

Transformation and positive cloning identification: a tube containing 50 μl of DH5α competent cells was placed on melting ice and lightly shaken to suspend the cells. Then, we added 10 μl of reaction fluid. This solution was shaken lightly and incubated for 30 min on ice. The solution was then placed into a 42°C water bath for 90 s to initiate heat shock. After this, it was quickly put on ice for 2 min. We added 700 μl of LB liquid medium and then incubated at 37°C for 60 min. We then centrifuged the sample at 5000 *g* for 1 min to collect the bacteria. A certain amount of bacteria was evenly applied to a plate containing streptomycin antibiotics. This was then coated with sterilized glass beads. After the bacterial solution was absorbed by the agar, it was inverted at 37°C overnight. We then selected colonies for PCR positive cloning identification and detection of primers for forward (PF) and primer 4.

*Agrobacterium* strain GV3101 transformation: We used the Plasmid Mini Kit I (D6943, Beijing Think-Far Technology Co. Ltd., Beijing, China) to extract the right sequencing plasmid. We placed GV3101 competent cells on ice, added 5–10 μl of plasmid, and slightly shook the solution. We then placed it in an ice bath for 10 min, froze it in liquid nitrogen for 5 min, placed it in a 37°C water bath for 5 min, and then an ice bath for 5 min. Then, we mixed it with 800 μl of antibiotic-free LB liquid medium, shook it at 28°C for 3–4 h, and centrifuged it at 5000 rpm for 1 min. We applied an appropriate amount of bacteria onto a plate (containing spectinomycin and rifampicin), inverted and cultured at 28°C. After a single colony grew out, we selected six single colonies per gene for colony PCR verification. The detection primers were the same as those used with the amplified gene primer.

Transient transformation of tobacco to subcellular localization: the tested *Agrobacterium* strain GV3101 solution was centrifuged (200 rpm) at 28°C overnight. We then added 1.5 ml of bacteria solution to a sterile centrifuge tube and centrifuged at 8000 rpm for 2 min. Room temperature 20°C precipitated the bacteria solution, suspending the bacteria. A small amount of suspended bacteria solution was diluted 10 times and then valued OD_600_. We multiplied the OD_600_ by 10 to obtain the suspended bacterial solution’s OD_600_ value. Finally, we calculated the suspension solution for infection as 0.5–5 μl, and the OD_600_ value as 0.4. We placed the final suspension solution in a 1.5 ml centrifuge tube and set it at 20°C for 1–3 h to initiate infection. Before infection, we placed the tobacco samples under a white fluorescent lamp for 1 h to open pores. A sample of three inverted leaves and another sample of four inverted leaves were selected for the infection experiments (infection between two veins). Two leaves were selected from one plant and infected with one bacterial solution. We gently rubbed the back of a leaf blade (0.5 cm^2^) with a syringe (without the needle) or punctured it with a small needle to remove the waxy layer. A suspension of 1 ml was allowed to absorb into each tobacco leaf to initiate infection. Then, we put the samples back into a culture room for the night. The expression was highest 2–3 days after infection. The infection area was cut, and the epidermis was peeled under a fluorescence microscope. Samples were examined using laser confocal microscopy (FV3000, Olympus, Tokyo, Japan), and excitation of fluorescence was performed at 488 nm for green fluorescent protein (GFP) fluorescence and 561 nm for chlorophyll fluorescence and bright field, and emission setting was 500–550 nm for GFP fluorescence and 570–620 nm for chlorophyll fluorescence and bright field.

### Expression Analysis of Sucrose Metabolism-Related Genes

Gene expression analysis was performed using qRT-PCR. Total RNA was extracted from roots, stems, leaves, and fruits of goji berry using RNAprep Pure Plant Kit (Tiangen, Beijing, China) according to the manufacturer’s instructions. RNA quality was assessed by performing electrophoresis on 0.8% agarose gel, and RNA concentration was measured using a NanoDrop 2000 spectrophotometer (Thermo Scientific, United States). Quantitative PCR (qPCR) was performed with the Lightcycler 480 system (Roche). Specific qPCR primers for *LBAI*, *LBSS*, *LBSPS*, *LBNI*, and β-actin (internal reference) were designed based on a full cDNA sequence (SupplementaryTable S2) using the PrimerQuest Tool.^6^ First-strand reverse transcription was performed using the miRcute Enhanced miRNA cDNA First Strand Synthesis kit (KR211, Tiangen, Beijing, China), prepared as follow: 10.0 μl 2 × miRNA RT Reaction Buffer, total RNA, 2.0 μl miRNA RT EnzymeMix, and up to 20 μl RNase Free dH_2_O. Reverse transcription was performed at 42°C for 30 min, followed by 2 min at 95°C to inactivate the enzyme, and the temperature was then decreased to 4°C. Subsequently, qPCR was performed using the miRcute Plus miRNA qPCR Detection kit (SYBR Green; FP411, Tiangen, Beijing, China). The reaction volume of 20 μl consisted of 10 μl 2 × miRcute Plus miRNA Premix, 0.4 μl PCR forward (10 μM) and reverse (10 μM) primers, 1.0 μl cDNA template, and 8.2 μl dH_2_O. The reaction program for qRT-PCR was denaturation at 95°C for 15 min, followed by 45 cycles of 94°C for 30 s, and 60°C for 1 min. Real-time PCR was performed for each gene using three biological replicates and three technical replicates. Relative expression levels of *LBAI*, *LBSS*, *LBSPS*, and *LBNI* were calculated using the 2^−ΔΔct^ method with three technical replicates (Livak and Schmittgen, 2001).

### Statistical Analysis

The observed results of chlorophyll, photosynthesis, sugar content, sucrose metabolism-related enzyme activities, and qPCR data were analyzed by one-way ANOVA model using GraphPad prims software (version 7, GraphPad Software Inc, La Jolla, CA, United States), with Tukey’s HSD multiple comparisons applied at a 0.01 significance level. Pearson analysis was applied to identify the correlation among sugars in leaves and sugars and enzymes in berries.

## Results

### Chlorophyll and Photosynthesis

**Figure 2:**
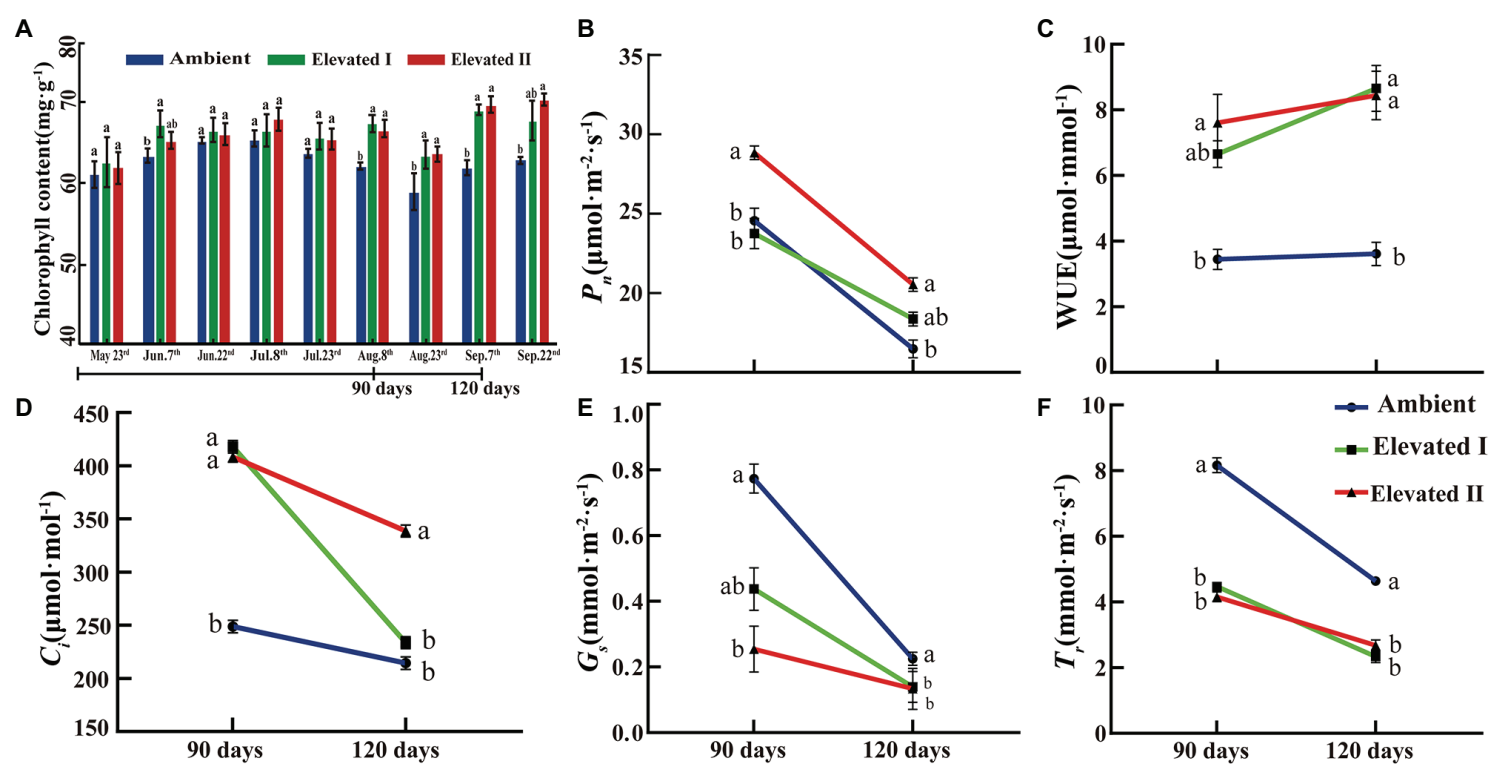
Chlorophyll content **(A)** and photosynthetic capacity **(B–F)** under elevated and ambient CO_2_ in goji berry for 90 and 120 days. *Pn*, net photosynthetic rates, WUE, water use efficiency, *Ci*, intercellular CO_2_ concentration, *gs*, stomatal conductance, *Tr*, transpiration rate. Different lowercase letters indicate significant difference at *p* < 0.05.

Leaf chlorophyll content was determined under ambient and eCO_2_ conditions at 15-day intervals during the growth period of goji berry seedlings. As shown in Figure 2A, chlorophyll content increased over time under different eCO_2_ concentrations, and significant (*p* < 0.05) differences were observed on five dates (June 7th,August 8th and 23rd, and September 7th and 22nd).

Photosynthetic parameters, including *Pn*, *C_i_*, *g_s_*, and *Tr*, were measured. Additionally, WUE was estimated to evaluate CO_2_ treatment effects on photosynthesis and biomass accumulation at 90 and 120 days after treatment initiation. *Pn* (Figure 2B), *C_i_* (Figure 2D), *g_s_* (Figure 2E), and *Tr* (Figure 2F) showed a consistent downward trend at 90 and 120 days under eCO_2_. In contrast, WUE (Figure 2C) was significantly (*p* < 0.05) higher in plants grown under eCO_2_ (at both I and II) after 90 or 120 days than in plants exposed to ambient CO_2_.

### Sugar Content and Sucrose Metabolism-Related Enzyme Activities

**Figure 3:**
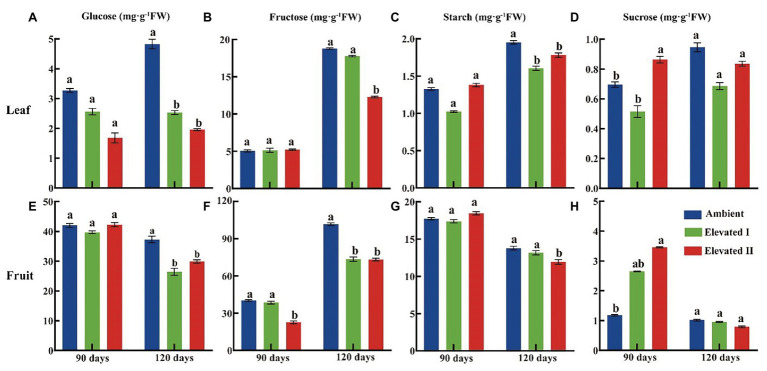
Glucose **(A,E)**, fructose **(B,F)**, starch **(C,G)**, and sucrose **(D,H)** content in leaf and fruit under elevated and ambient CO_2_ for 90 and 120 days. Different lowercase letters indicate significant difference at *p* < 0.05. FW, Fresh weight.

**Figure 4:**
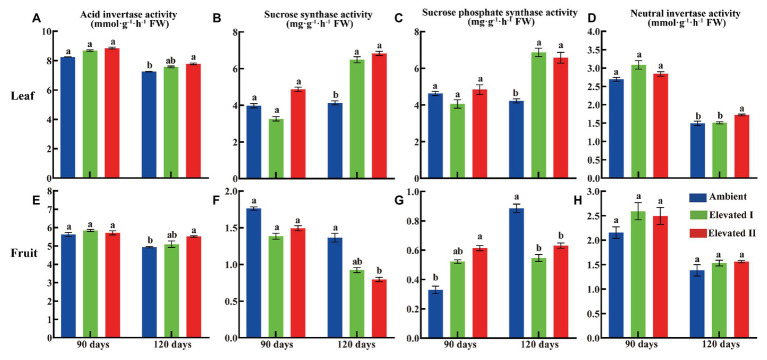
Activity of acid invertase **(A,E)**, sucrose synthase **(B,F)**, sucrose phosphate synthase **(C,G)**, and neutral invertase **(D,H)** in leaf and fruit under elevated and ambient CO_2_ for 90 and 120 days. Different lowercase letters indicate significant difference at *p* < 0.05. FW, Fresh weight.

Various sugar components and sucrose metabolism-related enzyme levels were determined in goji berry leaves and fruits grown under ambient and eCO_2_ for 90 and 120 days (Figures 3, 4). All four sugars under study – glucose, fructose, sucrose, and starch – followed a downward trend under eCO_2_. Glucose, fructose, and starch levels decreased significantly (*p* < 0.05) in leaves and fruits grown under elevated II eCO_2_ for 120 days (Figures 3A−C,E−G), whereas sucrose increased significantly (*p* < 0.05) in 90 day samples (Figures 3D,H) compared with plants under ambient CO_2_ conditions. The activity of all four sucrose metabolism-related enzymes – LBAI, LBSS, LBSPS, and LBNI – were also determined both at 90 and 120 days (Figure 4). These four enzymes showed an upward trend in leaves under eCO_2_, and this trend was significant (*p* < 0.05) under eCO_2_ II in 120 day samples (Figures 4A–D). However, compared with plants grown under ambient conditions, plants under eCO_2_ II showed significantly (*p* < 0.05) reduced LBSS and LBSPS activities and increased LBAI activity after 120 days.

### Correlation Between Sugar Content and Sucrose Metabolism-Related Enzyme Activities

**Figure 5:**
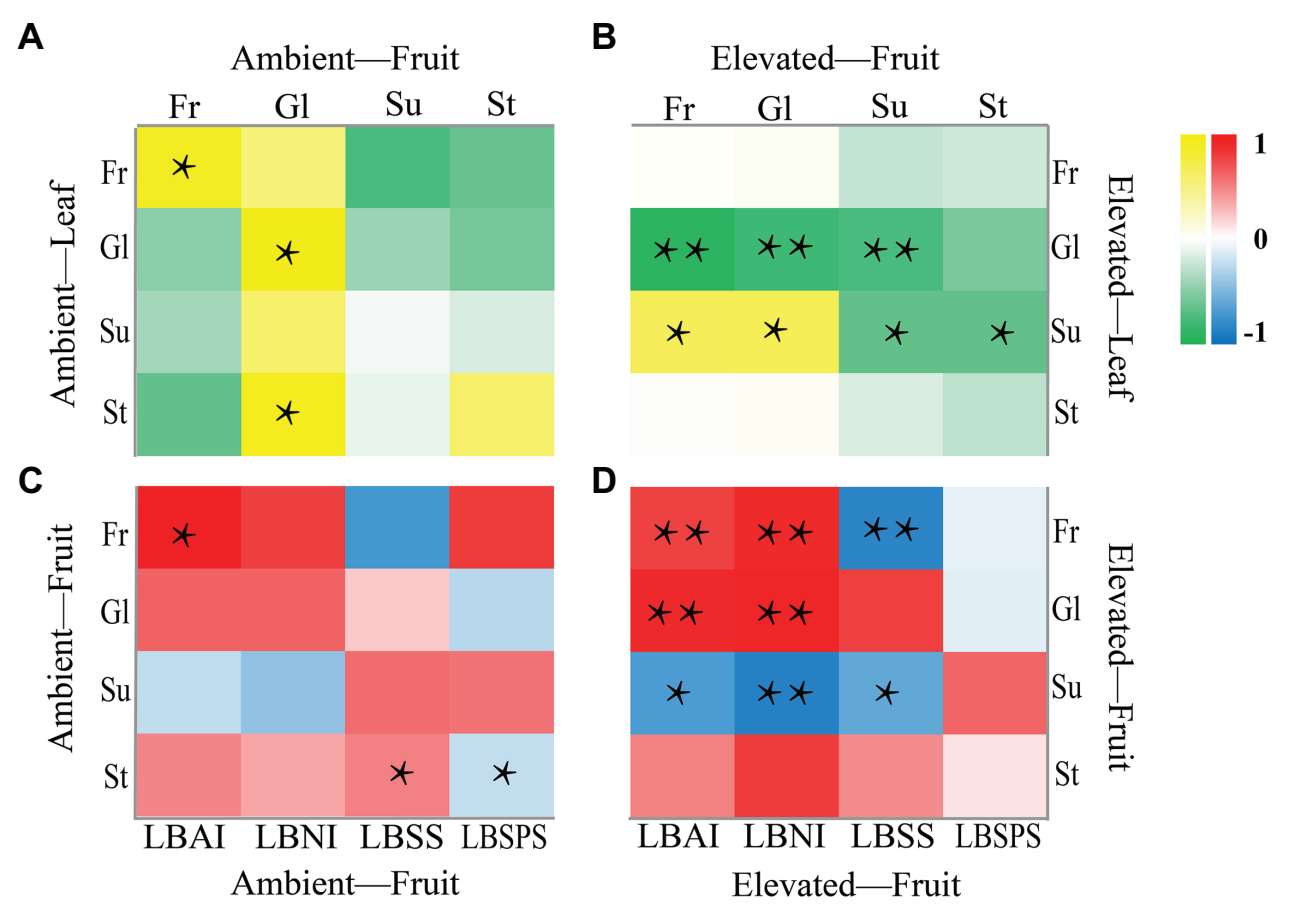
Correlation analysis of sugar in leaf and fruit **(A,B)** and sugar and enzyme in fruit **(C,D)**. ^*^p < 0.05, ^**^p < 0.01.

Pearson correlation analysis was used to analyze the correlation between sugars in leaves and between sugars and enzymes in berries (Figure 5). A positive, significant (*p* < 0.05) correlation was observed between sucrose in leaves, and fructose (Pearson 0.677) and glucose (Pearson 0.683) in berries (Figure 5B); conversely, a strong (*p* < 0.01) negative correlation was detected between glucose in leaves and fructose (Pearson −0.901), glucose (Pearson −0.779), and sucrose (Pearson −0.730) in berries (Figure 5B). Furthermore, eCO_2_ enhanced a significant correlation between sugar and enzyme activities in fruit samples (Figures 5C,D). A positive, significant (*p* < 0.01) correlation was observed between LBAI and fructose (Pearson 0.758), LBAI and glucose (Pearson 0.874), LBNI and fructose (Pearson 0.882), LBNI and glucose (Pearson 0.901; Figure 5D); conversely, a significant negative correlation of LBAI and sucrose (*p* < 0.05, Pearson −0.689), LBNI and sucrose (*p* < 0.01, Pearson −0.868), LBSS with fructose (*p* < 0.01, Pearson −0.846), and sucrose (*p* < 0.05, Pearson −0.604) was observed in berries (Figure 5D).

### Sucrose Metabolism-Related Gene Identity, Molecular Cloning, and Vector Construction and Transformation

The sequences of the four sucrose metabolism-related genes were based on a previous transcriptome profiling study of goji berry (Ma et al., 2019a). A *BLAST* search was performed *via* the NCBI database against the transcriptome database of goji berry, and sucrose metabolism-related genes *LBAI*, *LBSS*, *LBSPS*, and *LBNI* were identified. A 2,808-bp *LBAI* (GenBank accession no. MN718195) sequence from the transcriptome shared 99% (KM191309.1) and 92% (KC776575.1) homology with the published sequence of goji berry, while a 3,288-bp *LBSS* (GenBank accession no. MN718196) sequence shared 99% homology with the corresponding sequence (KC907702.1 and KM191310.1); in turn, a 3,612-bp *LBSPS* (GenBank accession no. MN718196) and a 3,842-bp *LBNI* (GenBank accession no. MN718198) shared 99% (KM191308.1) and 84% (KR026955) homology with the corresponding sequences, respectively.

**Figure 6:**
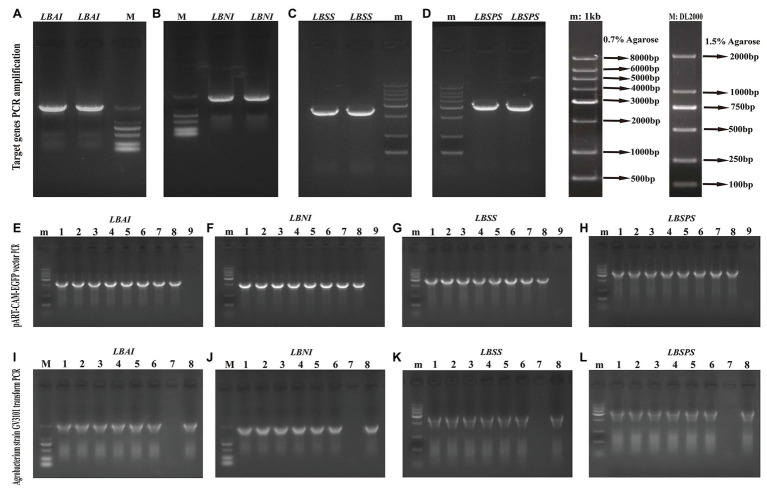
Target genes amplification **(A–D)**, vector pART-CAM-EGFP PCR identification **(E–H)**, *Agrobacterium* strain GV3101 transform PCR identification **(I–L)** of *LBAI*, *LBSS*, *LBSPS*, and *LBNI*, respectively. m: 0.7% agarose, 1 kb Marker; M: 1.5% agarose, DL2000 Marker; **(E–H)**: 1–8: single colony of *Escherichia coli* strain, 9: ddH_2_O negative control; **(I–L)**: 1–6: single colony of *Agrobacterium* strain, 7: ddH_2_O negative control, 8: plasmid pART-CAM-EGFP, positive control.

We identified the sucrose metabolism-related genes *LBAI*, *LBSS*, *LBSPS*, and *LBNI* by designing a primer (Supplementary Table S2) to amplify the target gene *ORF* sequence (Figures 6A–D). Then, we constructed the pART-CAM-EGFP plasmid and verified it using double enzyme digestion and further sequencing (Figures 6E–H) to verify that the sequence alignment was in line with the sequences of *LBAI*, *LBSS*, *LBSPS*, and *LBNI* (Supplementary Figure S1). After sequence verification, the recombinant plasmid pART-CAM-EGFP was transformed into the *Agrobacterium* strain GV3101 and validated using colony PCR (Figures 6I–L).

### Sucrose Metabolism-Related Gene Characterization and Subcellular Localization

**Figure 7:**
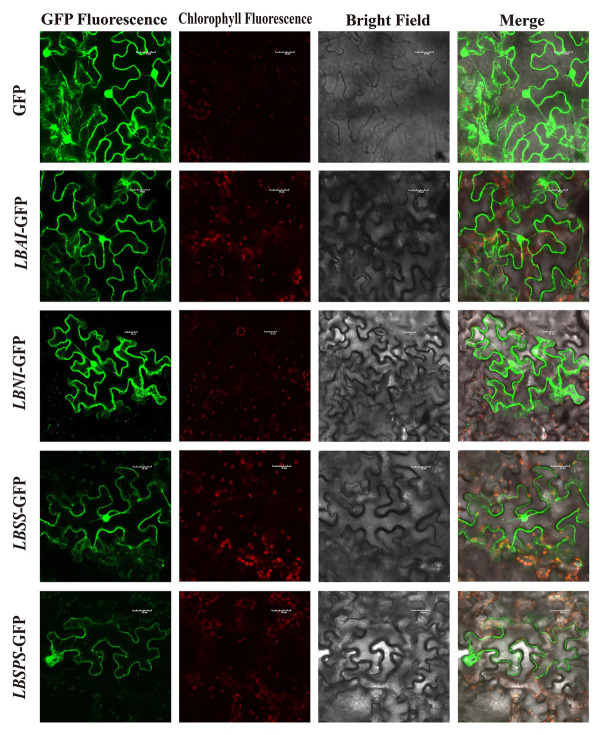
Subcellular localization of the *LBAI*, *LBSS*, *LBSPS*, and *LBNI* green fluorescent protein (GFP) in tobacco epidermal cells. GFP, transient expression of empty vector GFP; *LBAI-*GFP, transient expression of *LBAI-*GFP vector; *LBNI-*GFP, transient expression of *LBNI-*GFP vector; *LBSS-*GFP, transient expression of *LBSS-*GFP vector; *LBSPS-*GFP, transient expression of *LBSPS-*GFP vector.

Sequence analysis showed that *LBAI* contains 1,920 bp from *ORF*, which codes for 639 amino acids. In addition, *LBSS*, *LBSPS*, and *LBNI* comprise 805, 1,060, and 331 amino acids coded for by 2,418, 3,183, and 996 bp from *ORF*, respectively. Protein sequence analysis demonstrated that *LBAI* and *LBNI* contain a conservative structure domain belonging to the glycosyl hydrolases (Glyco_hydro) family. Similarly, both *LBSS* and *LBSPS* have sucrose synthase (Sucrose_synth) and glycosyltransferase (Glycos_transf) family domains (Supplementary Table S1). The physicochemical properties of proteins, such as formula, molecular weight, theoretical pI, instability index, aliphatic index, and grand average hydropathicity, were analyzed and listed in Supplementary Table S1. Moreover, prediction of protein secondary and tertiary structures (Supplementary Figure S2) indicated that *LBAI*, *LBSS*, *LBSPS*, and *LBNI* are mainly composed of alpha-helix, random coils, and extended strands (Supplementary Table S1). Besides this, subcellular localization prediction disclosed that *LBAI*, *LBSS*, *LBSPS*, and *LBNI* have the highest possibility of being located in the plasma membrane, mitochondrial matrix space, nucleus, and cytoplasm (Supplementary Table S1). Analysis indicated that *LBAI*, *LBSS*, *LBSPS*, and *LBNI* are soluble and non-secretory proteins synthesized in the cytoplasm without protein translocation abilities. To validate this prediction, the fusion of GFP pART-CAM-EGFP plasmid constructs were generated and temporarily expressed in tobacco mesophyll cells to examine their subcellular localization. The tobacco epidermal cells that were transformed with an empty GFP vector displayed fluorescence throughout the cell. The green fluorescence was distributed in the cytoplasm, nucleus, and plasma membrane. Similar to the control group, *LBAI*-GFP, *LBNI*-GFP, and *LBSS*-GFP were all found to be present in the nucleus, plasma membrane, and cytoplasm (Figure 7). These findings were largely consistent with the predictions from above bioinformatics. However, the only difference was that *LBSPS*-GFP was detected in the plasma membrane (Figure 7).

### Sucrose Metabolism-Related Gene Expression Patterns

**Figure 8:**
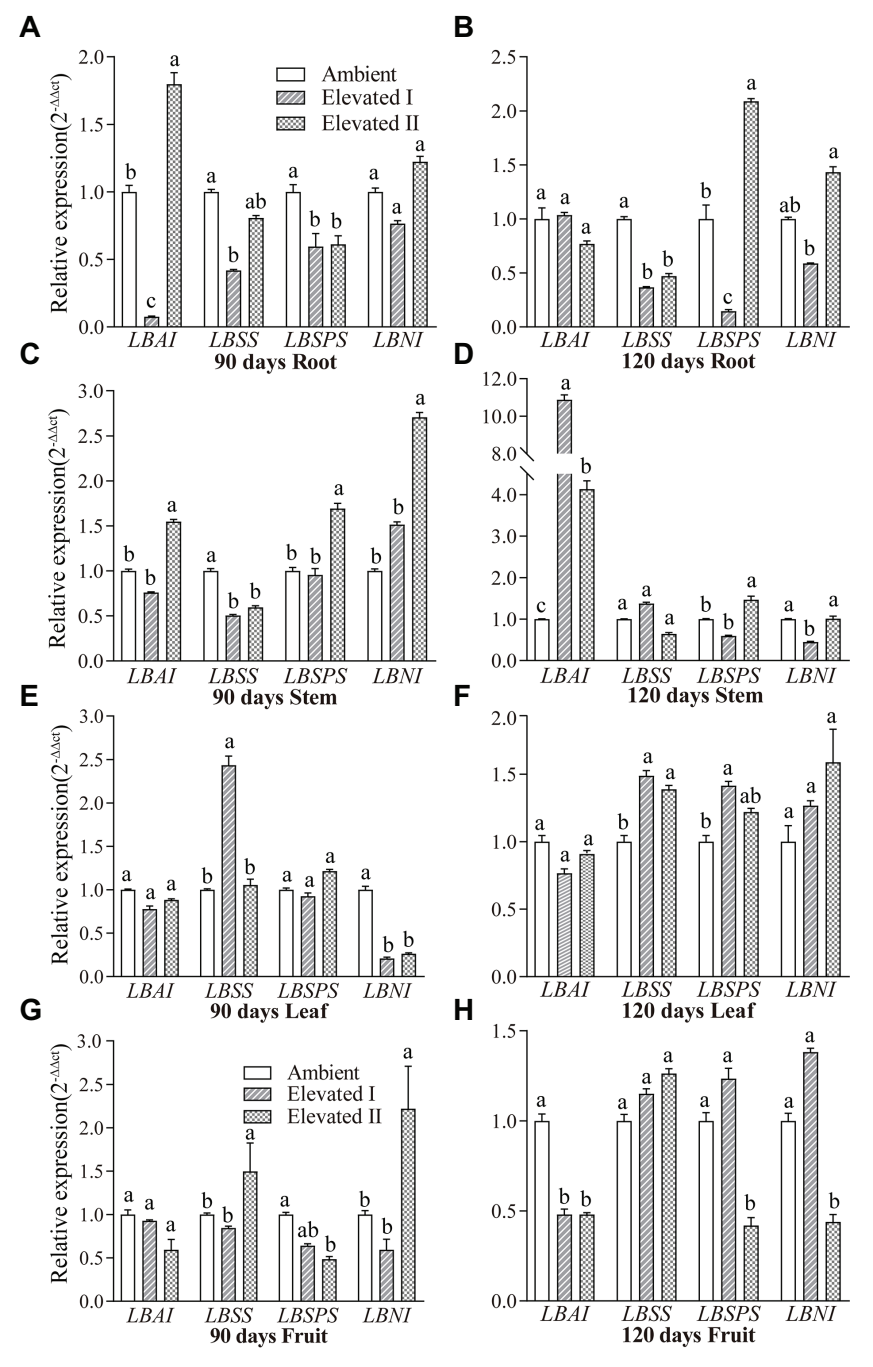
Expression patterns of gene *LBAI*, *LBSS*, *LBSPS*, and *LBNI* in root **(A,B)**, stem **(C,D)**, leaf **(E,F)**, and fruit **(G,H)** under elevated and ambient CO_2_ for 90 and 120 days. Different lowercase letters indicate significant difference at *p* < 0.05.

Sucrose metabolism-related gene expression was analyzed using qRT-PCR in different tissues of plants grown under eCO_2_ conditions (Figure 8). *LBAI*, *LBSS*, *LBSPS*, and *LBNI* were expressed in all tissues used in the study (root, stem, leaf, and fruit tissues). *LBAI* expression increased (*p* < 0.05) in roots and stems after 90 days (elevated II) and in stems after 120 days (Figures 8A,C,D), whereas *LBSS* increased (*p* < 0.05) in leaf after 90 and 120 days and in fruit after 90 days (Figures 8E–G). Additionally, *LBSPS* increased (*p* < 0.05) in stem after 90 days (elevated II), as well as in root and stem (elevated II), and leaf (elevated I) tissues after 120 days (Figures 8B–D,F). Moreover, *LBNI* increased (*p* < 0.05) in the stem, and fruit (elevated II) tissues after 90 days and in fruit (elevated I) tissues after 120 days (Figures 8C,G,H).

## Discussion

Previous studies showed that eCO_2_ had a remarkable impact on the growth and development of goji berry plants and on the accumulation of photosynthetic products (Cao et al., 2011; Guo et al., 2019). Long-term CO_2_ enrichment affected the allocation and accumulation of photosynthetic products in different organs. Based on observations on goji berry growing in OTCs over the entire growth period, eCO_2_ contributed to increasing vegetative growth, significantly increasing plant biomass and clearly changing fruit morphological traits (Ha et al., 2019). Additionally, compared to ambient CO_2_, eCO_2_ enhanced the photosynthetic capacity and reduced sugar and secondary metabolite levels; furthermore, long-term eCO_2_ treatment reduced sucrose synthesis and metabolism-related enzyme activity and affected the level of expression of genes involved in regulating sugar metabolism pathways (Liu et al., 2016; Ma et al., 2019a). However, although those studies revealed some physiological characteristics and molecular regulation mechanisms of goji berries under eCO_2_, to our knowledge, the study reported herein is the first one to focus on the relationship between different sugars and sucrose metabolism-related enzyme activities and the spatial-temporal expression pattern of the associated sucrose metabolism-related genes.

Previous studies found that short-term CO_2_ (30 or 60 days) treatments showed no significant differences in plant morphological and physiological characteristics (Cao et al., 2011; Hou and Cao, 2011; Liu et al., 2016; Guo et al., 2019; Ha et al., 2019). However, after 120 days, total sugars and carotenoid contents increased significantly, whereas flavone content decreased significantly. Transcriptome analysis of fruit has also shown that three genes play a key role in the regulation of four sugar metabolism pathways (Ma et al., 2019a). A previous study found that heights and ground-level diameters were significantly higher in plants grown in eCO_2_ compared to those grown in ambient conditions for 90 days. Additionally, fruit diameters and single fruit weights also significantly increased, whereas leaf areas increased after 60 days and were significantly different after 90 days (Ha et al., 2019). Hence, in our study, we chose to use 90 and 120 day CO_2_ treatments to further aid research in this field.

### Physiological Response to eCO_2_

Atmospheric CO_2_ is the substrate for photosynthesis. Unsurprisingly, changes in CO_2_ concentrations usually directly affect photosynthetic production and growth. Indeed, a high CO_2_ concentration enhances plant biomass by promoting photosynthesis and, consequently, boosting carbon assimilation (Kull et al., 2003; Bergamaschi et al., 2018). The present study observed a significant increase in chlorophyll contents after 90 days in both eCO_2_ treatments compared to ambient conditions. Concomitantly, *Pn*, WUE, and *C_i_* increased, whereas *g_s_* and *Tr* decreased in eCO_2_-treated goji berry plants. This effect was greater under the higher CO_2_ concentration, which is consistent with several studies. For example, a synchronous increase in *Pn*, *C_i_*, and WUE was observed in *Paris polyphylla* cultivars under eCO_2_ concentrations (800 μmol mol^−1^), while *g_s_* and *Tr* remained unchanged (Qiang et al., 2020). Different Indica rice cultivars consistently show an increase in *Pn* under eCO_2_ treatments but show no significant variations in leaf chlorophyll contents during short-term eCO_2_ treatments (Fabre et al., 2020). Furthermore, a previous study found no difference in photosynthetic rates between *Triodia pungens* plants grown in ambient and eCO_2_ concentrations; in this case, eCO_2_ resulted in lower *g_s_* and *Tr*, improved WUE, and higher dry biomasses (Xian et al., 2020). However, studies have also shown that plants often show photosynthetic downregulation under long-term eCO_2_ exposure, while biomass production may change and even decrease due to species-specific responses to different treatment durations (Reich et al., 2018; Mehta et al., 2019). In C3 plants, eCO_2_ concentrations result in reduced rubisco concentrations, and both photosynthesis and photorespiration decrease to maintain substrate concentrations (Luo et al., 2009). Studies have shown a significant increase in chlorophyll content and photosynthetic rate in yellow poplar (*Liriodendron tulipifera*) treated with eCO_2_ for 4 months (Je et al., 2018). Similarly, in the present study, long-term eCO_2_ treatment increased chlorophyll contents and *Pn*, thereby enhancing the photosynthetic ability of goji berries and leading to a greater accumulation of photosynthetic products.

The warming effect of globally elevated CO_2_ is also of concern, as CO_2_ and temperature have been shown to have interactive effects on some crops (Xu et al., 2013; Dwivedi et al., 2017). Previous studies showed that the morphological and physiological characteristics of goji berries change significantly under the stress of elevated temperature and drought. Using OTCs to simulate an atmospheric temperature increase of 3.7°C above ambient temperatures, seedling heights, ground-level diameters, leaf fresh weights, leaf dry weights, and leaf areas of goji berry were found to increase significantly. After 60 and 90 days of treatment, net photosynthesis rates (*Pn*) had decreased, but after 120 days, it had increased. In terms of fruit quality under elevated CO_2_ concentration treatment, after 90 days, the contents of carotenoids and flavones decreased significantly, but after 120 days, flavone content had increased (Ma et al., 2019a).

### Sugar Contents and Sucrose Metabolism-Related Enzyme Activities

Fruit and leaf glucose, fructose, starch, and sucrose contents decreased in the long-term eCO_2_ treatment in this study. Concomitantly, eCO_2_ affected sugar metabolism-related enzymes, increasing LBAI, LBSS, LBSPS, and LBNI in leaves while reducing LBSS and LBSPS in fruit tissues. Our previous work found that eCO_2_ reduces sugar and secondary metabolite levels (Cao et al., 2011; Ma et al., 2019a). Furthermore, we found that LBAI, LBSS, and LBSPS activities increase in fruit tissue after 90 days but decrease after 120 days of eCO_2_ treatment (Liu et al., 2016). Sucrose, glucose, and fructose synthesized in source tissues are the three major soluble sugars, sucrose is then transported to the sink tissues that contribute to total sugar content. Furthermore, these sugars essentially determine the number and quality of fruits produced (Tian, 2003; Basson et al., 2010). Sucrose metabolism-related enzymes including SS, SPS, NI, and AI, have all been found to play key roles in the assimilation and transport of sugars in plants (Zhang et al., 2012,^ 2019^). SPS is involved in sucrose synthesis, while AI and NI are involved in sucrose decomposition, and SS catalyzes either sucrose synthesis or catabolism (Liu et al., 2011).

In this study, LBAI, LBSS, LBSPS, and LBNI activities tended to increase in leaves collected after 90 days of treatment, although no significant differences relative to those of the control were observed. Similar results were observed in fruit tissues, except that LBSPS increased significantly in this case. LBSS and LBSPS play a pivotal role in sucrose biosynthesis and transport, and LBSPS activity increases in leaves under eCO_2_, thereby causing increased leaf sucrose contents (Hussain et al., 1999; Moore et al., 1999). However, after 120 days under eCO_2_, the activities of the above-mentioned four sucrose metabolism-related enzymes increased significantly in leaves, whereas in fruit tissues, LBSS and LBSPS decreased significantly. Simultaneously, LBAI increased and LBNI showed no change.

Sucrose and starch are the major end products of photosynthesis in most plants and they are also major carbon sources involved in the synthesis of other important structural and metabolic compounds, such as cellulose and proteins (Annunziata et al., 2017; Xie et al., 2018; Ma et al., 2019a). Additionally, only two types of enzymes can cleave sucrose in plants: sucrose invertase, and sucrose synthase (SS; Lugassi et al., 2015).

The leaf is the main photosynthetic organ and the products of leaf photosynthesis are transported to sink organs mainly as sucrose. Furthermore, sucrose metabolism-related enzymes are the key enzymes facilitating the entry of sucrose into several metabolic pathways (Zhang et al., 2017). As eCO_2_ significantly affects enzyme activity, it, therefore, affects many metabolic processes, including photosynthesis, and nutrient uptake and assimilation (Hofmann et al., 2013). In this study, the activities of LBAI and LBNI in fruit and leaf tissues were similar to those in plants under either ambient or eCO2 conditions, while sucrose contents and LBSPS activities significantly increased in fruits after 90 days of eCO_2_ treatment. This indicates that the short-term eCO_2_ treatment increased LBSPS enzyme activity and, thus, facilitated sucrose accumulation in this organ. However, long-term eCO_2_ exposure reduced SS and SPS activities in fruits and leaves, while starch and sucrose contents increased in leaves and decreased in fruits, compared to those with the short-term treatment. These results clearly indicate that long-term eCO_2_ conditions are not conducive to sugar accumulation in goji berry fruit. Similar results were also observed in goji berry fruits under 120 days elevated CO_2_ treatment, where the content of polysaccharides and fructose was significantly reduced (Ha et al., 2019). As global warming intensifies, goji berry may suffer negative impacts. Breeding goji berry varieties that show good tolerances to elevated CO_2_ and temperatures are important for coping with climate change in the future.

Correlations between the contents of different kinds of sugars and the four sucrose metabolism-related enzymes in leaf and fruit tissues were further analyzed under ambient and eCO_2_ conditions. Similar studies have found significant positive correlations between fructose and LBAI in goji berry leaves and fruits (Zheng et al., 2009; Wang et al., 2014). Furthermore, several studies have also shown that LBSPS activity is negatively correlated with starch and positively correlated with sucrose in fruits (Wang et al., 2004; Zhang et al., 2004).

### Regulation and Function of Sucrose Metabolism-Related Genes

An early study determined that four sucrose metabolism enzymes (AI, NI, SS, and SPS) work together to balance sugar transport and deposition or utilization in citrus fruits (Lowell et al., 1989). A study also confirmed the effects of different sucrose metabolism-related enzymes on the accumulation of different sugars during tomato fruit development (Miron and Schaffer, 1991). Several recent studies have reported the activities and expression patterns of sucrose metabolism-related enzymes in different species. As shown by a study on pineapples, SPS and SS activities, and gene transcript levels gradually increased, while NI decreased during pineapple fruit development (Zhang et al., 2019). Another study also showed that homologous cloning of SPS and NI genes and transcription expression analysis of SPS, SS, and NI indicate that there are distinct patterns related to sugar accumulation and composition in pineapple fruits (Zhang et al., 2012).

Recent studies have described the effects of the expression and regulation of the sucrose metabolism-related enzyme genes *LBAI*, *LBSS*, *LBSPS*, and *LBNI* on sugar accumulation in goji berries. However, the mechanism underlying plant regulation of sugar metabolism in fruits during acclimation to eCO_2_ conditions remains unknown. The enzymes LBAI, LBSS and LBSPS are closely related to the sugar synthesis in goji berry and mainly involvement in starch and sugar metabolism pathways (Ma et al., 2019a, 2021), the genes *LBAI*, *LBSS*, *LBSPS*, and *LBNI* are expressed in different tissues and are involved in important sugar synthesis and regulation pathways influencing enzymes and metabolism. Our study identified and isolated four sucrose metabolism-related genes – *LBAI*, *LBSS*, *LBSPS*, and *LBNI* – based on the *de novo* transcriptome of goji berry (Ma et al., 2019a). BLAST searches indicated that the sequences of these genes had high homology with reported sequences for goji berry (Wang et al., 2019). In addition, subcellular localization analysis revealed that *LBAI*, *LBNI*, and *LBSS* were predominantly located in the nucleus, plasma membrane, and cytoplasm, while *LBSPS* was localized in the plasma membrane. Previous studies have reported that in goji berry, AI was localized in vacuoles (Wang et al., 2014), SPS in the nucleus (Wang et al., 2013), and NI in the cytoplasm (Wang et al., 2019), but these analyses were based on bioinformatics. In this study, the expression position of the sucrose metabolism-related gene protein was determined by predicting and further verifying the transient transformation of tobacco. Differential expression patterns among roots, stems, leaves, and fruits under different CO_2_ conditions revealed that the genes have diverse spatiotemporal expression patterns. At least one study found that SPS and NI expression levels correlated with their corresponding enzyme activities during goji berry fruit development, whereas those of AI and SS were not related to changes in enzyme activities in different cultivars (Wang et al., 2019). Our data further demonstrates that these four sucrose metabolism-related genes play an important role in sugar metabolism in goji berry plants. Further development of cultivars that can regulate sugar metabolism through molecular breeding methods is needed.

## Conclusion

This study confirmed that chlorophyll, photosynthesis, various sugars, and related enzyme activities in goji berry leaves and fruits were significantly affected by eCO_2_ after 90 and 120 days. Additionally, *LBAI*, *LBSS*, *LBSPS*, and *LBNI* were identified based on transcriptome profiling and the analysis of characteristic, subcellar locations, and expression patterns. Protein sequence analysis indicated that *LBAI* and *LBNI* contain a Glyco_hydro family conservative structure domain, and both *LBSS* and *LBSPS* have Sucrose_synth and Glycos_transf family domains. In addition, subcellular localization analysis found all three genes (*LBAI*, *LBNI*, and *LBSS)* in the nucleus, plasma membrane, and cytoplasm, while *LBSPS* was found in the plasma membrane. The four genes were expressed in all examined tissues treated with eCO_2_, including roots, stems, leaves, and fruits. Our results provide a sound theoretical basis for understanding sugar metabolism responses to changes in CO_2_ concentrations and provide candidate genes for the future breeding of goji berry plants containing high sugar and secondary metabolites materials.

## Data Availability Statement

The datasets presented in this study can be found in online repositories. The names of the repository/repositories and accession number(s) can be found at: https://www.ncbi.nlm.nih.gov/genbank/, MN718195; https://www.ncbi.nlm.nih.gov/genbank/, MN718196; https://www.ncbi.nlm.nih.gov/genbank/, MN718197; and https://www.ncbi.nlm.nih.gov/genbank/, MN718198.

## Author Contributions

BC and LS designed the experiments. YM, YX, and RH performed the experiments. YM analyzed the data and drafted the manuscript. BC revised the manuscript. All authors contributed to the article and approved the submitted version.

### Conflict of Interest

The authors declare that the research was conducted in the absence of any commercial or financial relationships that could be construed as a potential conflict of interest.

## Footnotes

^1^https://www.ncbi.nlm.nih.gov/genbank

^2^https://web.expasy.org/protparam/

^3^http://www.ncbi.nlm.nih.gov/gorf/gorf.html

^4^http://www.cbs.dtu.dk/services/SignalP/

^5^https://swissmodel.expasy.org/

^6^http://sg.idtdna.com/Primerquest/Home/Index
