# Supplementary material for: Effects of Elevated CO2 on Photosynthetic Accumulation, Sucrose Metabolism-Related Enzymes, and Genes Identification in Goji Berry (Lycium barbarum L.)
Source: Front Plant Sci. 2021 Mar 11;12:643555. doi: 10.3389/fpls.2021.643555 (PMC7991576; doi:10.3389/fpls.2021.643555)
Supplement: Supplementary file 1 [file Data_Sheet_1.docx]

Supplementary Material

# Supplementary Figures and Tables

## Supplementary Figures

(d)

(c)

(b)

(a)

**Supplementary Figure 1.** The gene *LBAI* (a), *LBNI* (b), *LBSPS* (c), *LBSS* (d) homology sequence of alignment


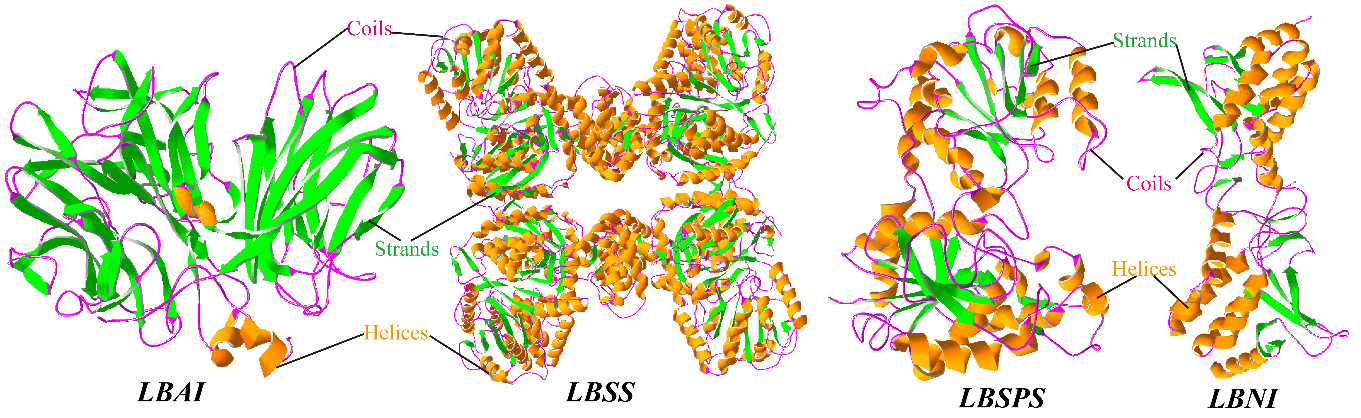


**Supplementary Figure 2.** The *LBAI*, *LBSS*, *LBSPS*, and *LBNI* protein spatial structure prediction.

| **Supplementary Table 1** Analysis of protein characteristics of genes *LBAI*, *LBSS*, *LBSPS, LBNI* | | | | | | | | | | | | | | |
| --- | --- | --- | --- | --- | --- | --- | --- | --- | --- | --- | --- | --- | --- | --- |
| **Gene** | **Sequence length(bp)** | **Protein length(aa)** | **Domains and position** | **Physicochemical properties** | | | | | |  | **Secondary structure** | | | **Subcellular location** |
|  |  |  |  | **Formula** | **Molecular weight (kDa)** | **Theoretical**  **pI** | **Instability index** | **Aliphatic index** | **Grand average of hydrophilicity(GRAVY)** |  | **Alpha helix (Hh)(%)** | **Random coil (%)(Cc)** | **extended strand (%)(Ee)** |  |
| *LBAI*  (MN718195) | 2802 | 639 | Glyco_hydro_32 (112-625) | C_3187_H_4869_N_839_O_950_S_14_ | 70.59 | 5.90 | 40.04 | 80.04 | -0.244 |  | 17.68 | 60.56 | 21.75 | Plasma membrane |
| *LBSS*  (MN718196) | 3288 | 805 | Sucrose_synth  (8-553)  Glycos_transf_1  (557-741) | C_4197_H_6543_N_1111_O_1195_S_26_ | 92.52 | 6.00 | 32.62 | 93.01 | -0.250 |  | 46.34 | 38.63 | 15.03 | Mitochondrial matrix space |
| *LBSPS*  (MN718197) | 3612 | 1060 | Glycos_transf_1 (464-649)  S6PP (767-1022)  Sucrose_synth  (161-439) | C_5239_H_8372_N_1486_O_1604_S_43_ | 119.22 | 6.19 | 45.80 | 85.92 | -0.460 |  | 40.28 | 44.91 | 14.81 | Nucleus |
| *LBNI*  (MN718198) | 3842 | 331 | Glyco_hydro_100 (93-286) | C_1652_H_2554_N_440_O_485_S_28_ | 37.24 | 5.22 | 56.40 | 80.76 | -0.029 |  | 46.83 | 46.53 | 6.65 | Cytoplasm |

| **Supplementary Table 2** List of primer sequences | | | |
| --- | --- | --- | --- |
| Gene name | Primer name | Primer sequence（5’-3’）RACE | Use of primers |
| *LBNI* | F | TTGGAGAGGACACGCTCGAGATGTCTACACCCTCTATG | Target gene amplification |
|  | R | CCCTTGCTCACCATGAATTCAAATGAATTTGATCTTTTC |  |
| *LBSPS* | F | TTGGAGAGGACACGCTCGAGATGGCTGGTAATGAATGGA |  |
|  | R | CCCTTGCTCACCATGAATTCTGTGCCTAATCTAGACACT |  |
| *LBSS* | F | TTGGAGAGGACACGCTCGAGATGGCTGAACGTGTACTGA |  |
|  | R | CCCTTGCTCACCATGAATTCCTCAACAGCCAATGGGACA |  |
| *LBAI* | F | TTGGAGAGGACACGCTCGAGATGGCTACCCACCATTCCAG |  |
|  | R | CCCTTGCTCACCATGAATTCCAAATTTTCCAAGGGGAAG |  |
| Primer 4 |  | GGTAGCGGCTGAAGCACTG |  |
| PF |  | AGAAGACGTTCCAACCACG |  |
| *LBAI* | F | CTAGTCCCTGTGGCATTGTT | qRT-PCR |
|  | R | GTCAAGGAGGAAGAACAGTCAT |  |
| *LBSS* | F | GTAAGACCGAAAGCCTCGTAAA |  |
|  | R | AGATGAACCGTGTGAGGAATG |  |
| *LBSPS* | F | TGTTCCTCTGCTTGCATCTC |  |
|  | R | CGTAATCCGTGTCTCCAGTTT |  |
| *LBNI* | F | GTCTTCCTCAAGTGCAACCA |  |
|  | R | GGGAAGCAAGCTCGGAAATA |  |
